# Hybrid Management of Giant Solitary Intracranial Fibrous Tumors: A Report of Two Cases

**DOI:** 10.7759/cureus.87798

**Published:** 2025-07-12

**Authors:** Diego Julian Alvis Peña, Jorge Antonio Morales Baez, Fernando Espinosa Lira, Melvin Francisco Ulerio Paniagua, Arturo Ayala Arcipreste

**Affiliations:** 1 Neurological Surgery, Hospital Juárez De México, Mexico City, MEX

**Keywords:** hemangiopericytoma, non-meningothelial mesenchymal tumors, pericytes, solitary fibrous tumor (sft), therapeutic embolization

## Abstract

Solitary fibrous tumors (SFTs) are rare tumors of vascular origin, derived from pericytes surrounding blood vessels. Their frequency of presentation is mainly in adults, and they can be in various parts of the body, such as the extremities, head, neck, retroperitoneum, and abdomen. These tumors can be either benign or malignant, and their behavior and clinical course are variable, with a significant risk of recurrence and metastasis. We present a two-case report: the first involving a 25-year-old man with right hemiparesis and a history of two prior surgeries at another institution for a diagnosed SFT; the second involving a 48-year-old man with decreased visual acuity and personality changes, with a biopsy confirming SFT. Both cases were managed with embolization followed by resection, with no residual tumor remaining. Treatment of SFTs with embolization and complete surgical resection improves surgical outcomes and reduces the risk of recurrence.

## Introduction

Solitary fibrous tumors (SFT) are rare tumors of vascular etiology, derived from pericytes surrounding blood vessels. Their frequency of presentation is mainly in adults, and they can be in many parts of the body, such as the extremities, head, neck, retroperitoneum, and abdomen. These tumors can be either benign or malignant, and their behavior and clinical course are variable, with a significant risk of recurrence and metastasis [1].

The prevalence and incidence of SFTs are difficult to determine accurately due to their low frequency. According to an analysis based on the SEER database, the incidence of TFS in 2016 was 0.060 per 100,000 individuals [1]. These tumors are rare and account for less than 1% of all intracranial tumors [1]. Furthermore, in the context of central nervous system (CNS) tumors, TFS are classified as non-meningothelial mesenchymal tumors [2,3].

In the pediatric population, SFTs are even rarer, accounting for approximately 10% of all cases [3]. The literature suggests that these tumors have a tendency for local recurrence and can metastasize to extraneural sites such as the lungs, bones, and liver [3,4]. Mortality associated with TFS varies according to location and treatment. In the case of intracranial SFTs, research by Rutkowski et al. reports a median survival of 13 years, with 1-, 5-, 10-, and 20-year survival rates of 95%, 82%, 60%, and 23%, respectively. Total tumor resection is associated with better survival rates compared to subtotal resection, especially when adjuvant radiotherapy is not used [4]. In another study, the 10-year mortality rate was observed to be 30% in a series of 106 cases, with recurrence and metastasis as the main causes of death [5]. Furthermore, TFS of the central nervous system has a 10-year survival rate of 59% [6,7].

Histologically, TFS are characterized by a distinctive vascular pattern of abundant spindle cells and stag-horn vascular branches, and their diagnosis is confirmed by histopathology and immunohistochemistry, differentiating them from other mesenchymal tumors [6,7]. The most common clinical presentation is headache (58%), followed by nasal symptoms (19%), ocular symptoms (10%), auditory symptoms (5%), facial palsy (5%), and pituitary abnormalities (2.43%); the main treatment is maximally safe surgical resection to minimize the risk of local recurrence [5,8]. The usefulness of adjuvant radiotherapy and chemotherapy is limited, and surgery remains the most effective treatment to improve survival [8].

Considering the above, these data underscore the importance of complete surgical resection to improve survival and the need for long-term follow-up due to the risk of recurrence and metastasis.

## Case presentation

We report two cases of SFT managed at Hospital Juárez de México, emphasizing preoperative embolization to reduce surgical risk, along with their clinicopathological characteristics.

Case 1

A 25-year-old male patient presented with right hemiparesis (grade 3/5), difficulty in ambulation, restricted range of motion, dysphasia, and a visible swelling with skin tension over the frontoparietal region. He had previously undergone two partial resections of a giant left frontoparietal tumor at another institution, with histopathology confirming a diagnosis of SFT/hemangiopericytoma, WHO Grade II. Subsequently, in September 2019, preoperative embolization was performed using an embolizing copolymer, achieving 100% devascularization via the M3 and M4 branches of the left middle cerebral artery (MCA), the left callosomarginal artery, the left middle meningeal artery, and the left superficial temporal artery. This was followed by a gross total resection of the tumor, without any additional neurological deficits. Two years later, in December 2022, the patient underwent cranioplasty with a PEEK plate. He is currently under treatment for epilepsy with oral levetiracetam 1 g every eight hours.

**Figure 1:**
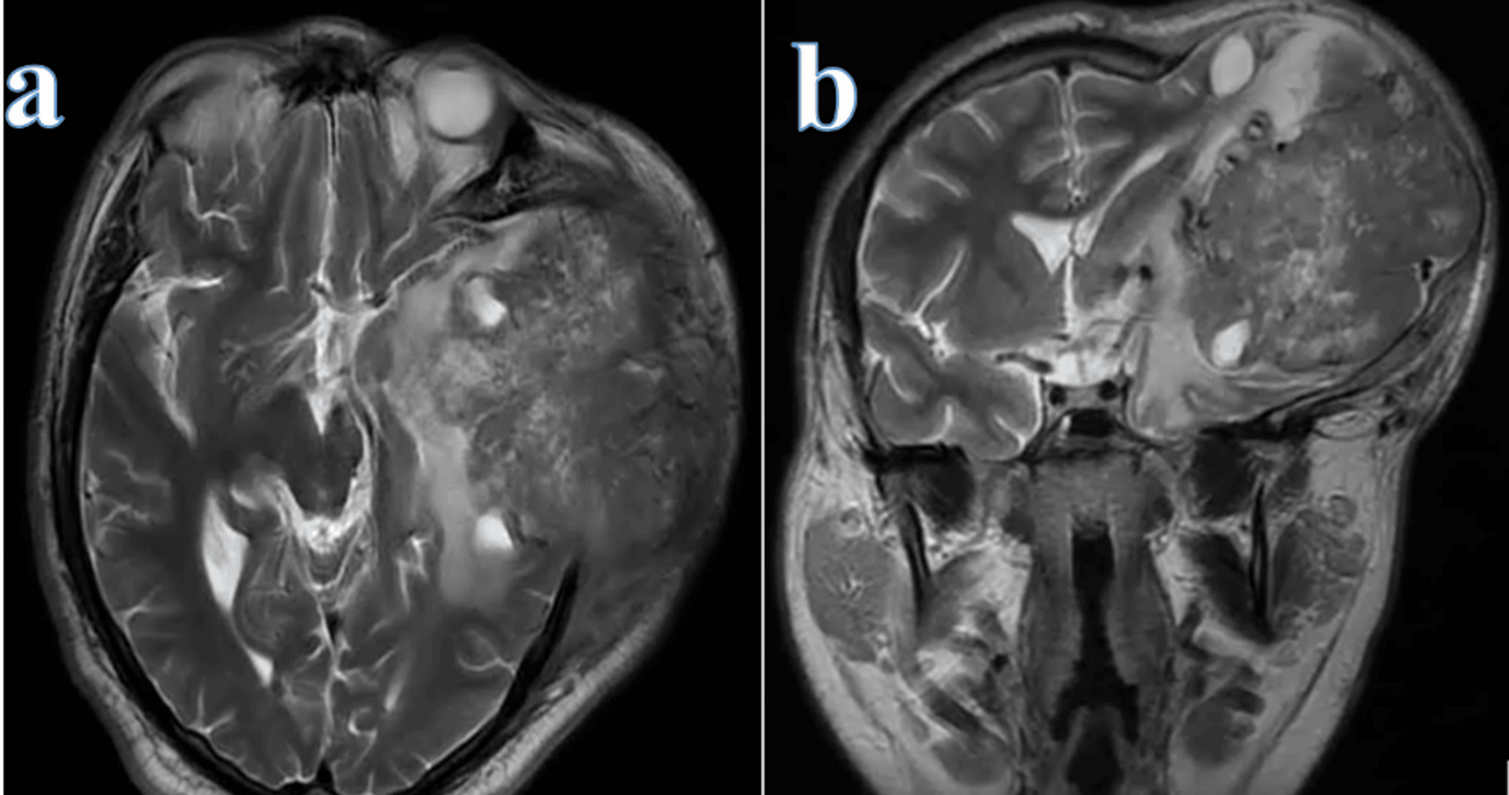
Preoperative MRI, 2019 T2-weighted MRI in axial (a) and coronal (b) sections showing a heterogeneous left-sided lesion with irregular margins, causing mass effect with midline shift, partial collapse of the ipsilateral ventricular system, and transcalvarial herniation.

**Figure 2:**
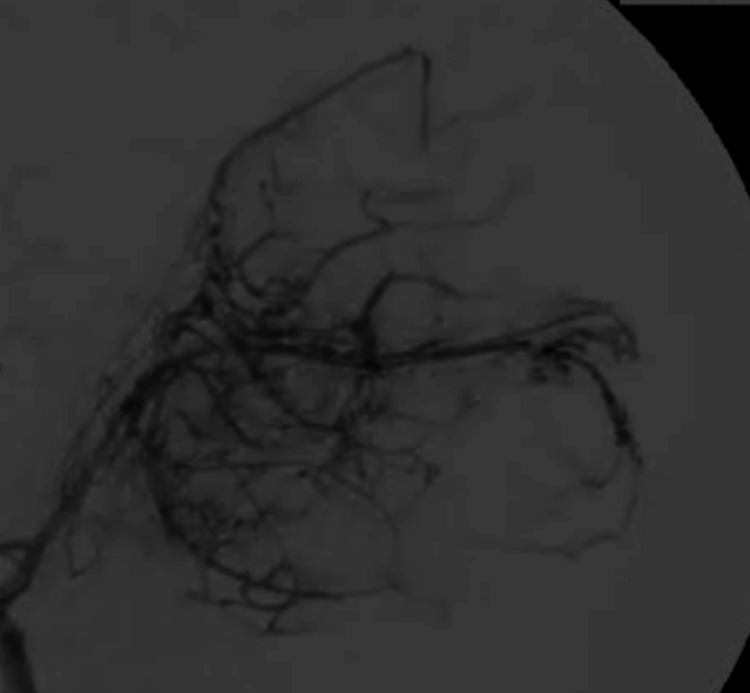
Therapeutic cerebral panangiography Findings showing 100% embolization through the M3 and M4 branches of the left middle cerebral artery (MCA), left callosomarginal artery, left middle meningeal artery, and left superficial temporal artery.

**Figure 3:**
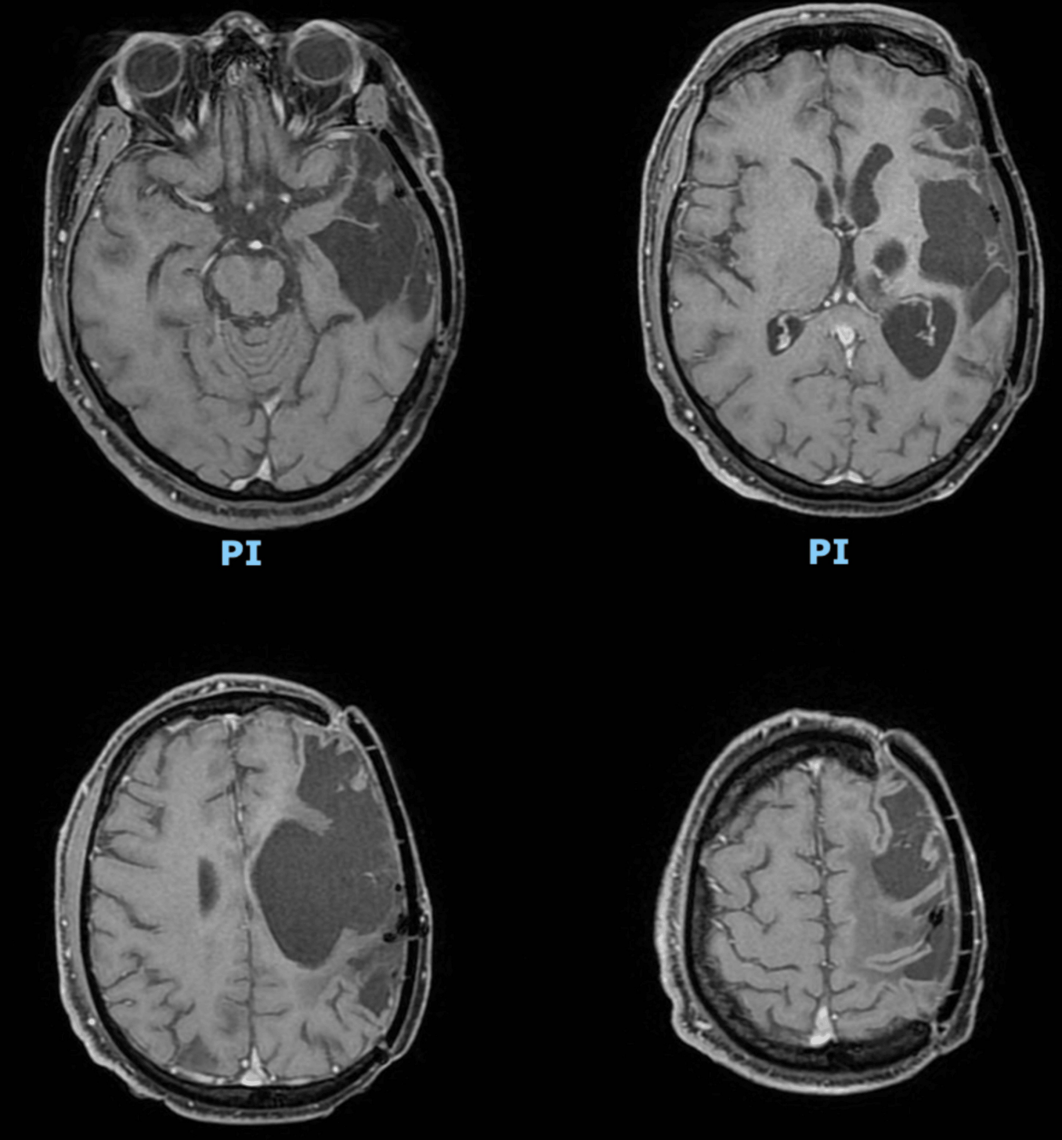
Postoperative contrast-enhanced MRI, 2024 In the axial section, no recurrent lesion is observed, and an area of encephalomalacia is present at the site of surgical resection.

Neurosurgical intervention was performed, including a new embolization of the SFT and maximal safe tumor resection via the previous surgical approach. Histopathological analysis confirmed a diagnosis of SFT/hemangiopericytoma, WHO Grade III. Postoperatively, the patient showed improvement in the range of motion of the right hand and recovery of memory. Outpatient follow-up with control imaging studies demonstrated no evidence of tumor recurrence (Figures 1, 2, 3).

Case 2

A 48-year-old male patient with a history of tumor resection at an external institution via a left orbitocraniotomy approach in March 2024 presented with a histopathological diagnosis of SFT, STAT6-positive, WHO Grade I. The procedure required transfusion of five units of packed red blood cells.

Upon admission to the neurosurgery department, the patient presented with hypertension and progressively worsening visual acuity, eventually reduced to counting fingers. He also exhibited personality changes, including emotional lability, social withdrawal, irritability, and periods of aggression.

Neurological examination revealed visual acuity of 20/25 in the right eye and amaurosis in the left eye, without visual field (campimetric) defects. Pupillary examination showed 2 mm in the right eye and 4 mm in the left eye, with a present photomotor reflex and a relative afferent pupillary defect (RAPD) in the left eye. Examination of the remaining cranial nerves revealed no abnormalities.

Surgical resection was performed in July 2024 following endovascular management via embolization. Angiographic findings revealed a left frontal lesion with vascular supply from the anterior cerebral and ophthalmic arteries. Approximately 85% of the left frontotemporal neoplastic lesion was embolized using three vials of EVOH. Intraoperatively, the lesion appeared white, arising from the anterior cranial fossa floor, with a fibrous consistency and moderate aspirability. A remnant of vascularized embolic copolymer was also noted. Histopathological examination described a spindle cell mesenchymal neoplasm with marked vascularity. Immunohistochemistry showed STAT6 positivity, heterogeneous nuclear features, CD34-positive vascular endothelium, and negative staining for SOX10 and S100. The Ki-67 index was positive in 10% of neoplastic nuclei, consistent with a diagnosis of SFT, WHO Grade I.

**Figure 4:**
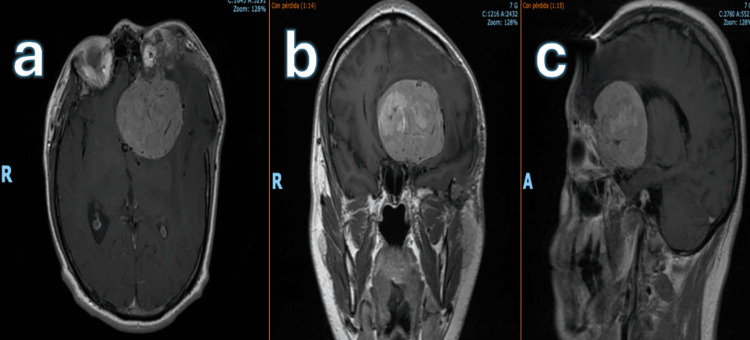
Preoperative MRI, 2024 T1-weighted MRI in axial (a), coronal (b), and sagittal (c) sections showing a heterogeneous lesion with regular margins in the left fronto-orbital region, causing mass effect with midline shift and partial collapse of both the ipsilateral and contralateral frontal horns of the ventricular system.

**Figure 5:**
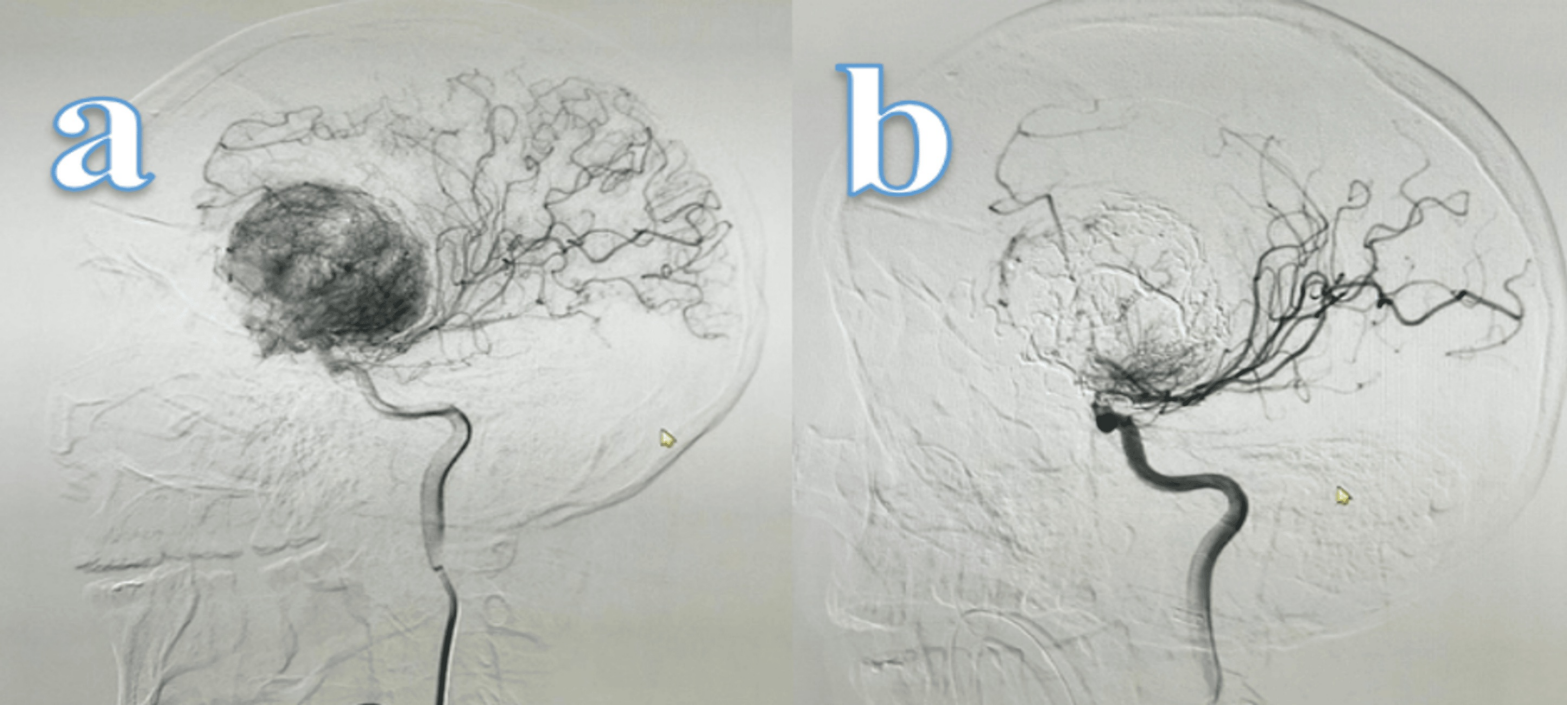
Therapeutic cerebral panangiography Pre-embolization (a) and post-embolization (b) images showing a left frontal lesion with vascular supply from the anterior cerebral artery and ophthalmic artery. Approximately 85% of the left frontotemporal neoplastic lesion was embolized using three vials of embolizing copolymer.

**Figure 6:**
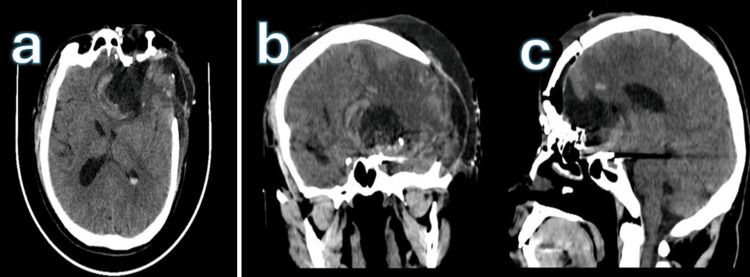
Postoperative non-contrast head CT scan, 2024 Axial (a), coronal (b), and sagittal (c) sections showing the left orbitozygomatic craniectomy site, with a small residual hyperdense lesion at the left anterior skull base and a hypodense area consistent with the surgical bed.

Postoperatively, the patient had a Glasgow Coma Scale score of 14, with disorientation to time and place and persistent emotional lability, including irritability and aggressiveness. Visual acuity was 20/20 in the right eye and amaurosis in the left, with a persistent afferent pupillary defect. No additional cranial nerve deficits were observed. Motor strength was 3/5 in the right upper limb, 4/5 in the right lower limb, and 5/5 throughout the left hemibody. No other positive findings were noted on neurological examination (see Figures 4, 5, 6).

## Discussion

Intracranial SFTs were first reported by Begg and Garrett in 1954. In general, SFTs are highly vascularized neoplasms with low prevalence and incidence, originating from mesenchymal spindle cells [1,6]. Intracranial SFTs of the central nervous system are rare, accounting for only 0.09-1% of all intracranial tumors. According to the SEER database, the incidence of intracranial SFTs in 2016 was 0.060 per 100,000 individuals. The 10-year mortality rate for SFTs can reach up to 30%, while the 10-year survival rate is approximately 59% [1,6,9]. From a pathophysiological standpoint, intracranial SFTs arise from pericytes located in capillary walls and postcapillary venules, as well as from mesenchymal spindle cells. These tumors are typically associated with high mitotic activity, contributing to their potential for local invasion, distant metastasis, and recurrence [6,10]. Their growth pattern and locations are similar to meningiomas, commonly occurring in the cerebral convexity, the falx cerebri, and the tentorium cerebelli [6,10,11]. Clinical symptoms usually manifest when the tumor reaches a significant size or is located in eloquent areas of the brain, leading to compression or invasion of functional brain regions. Symptoms may include headache, seizures, and sensorimotor deficits. In terms of frequency, reported symptoms are: headache (58%), nasal symptoms (19%), ocular symptoms (10%), auditory symptoms (5%), facial paralysis (5%), and pituitary abnormalities (2.43%) [11-13]. In our two clinical cases, patients presented with headache and ocular symptoms, findings that are consistent with those reported in the literature.

Radiologically, intracranial SFTs often resemble meningiomas. They appear as extra-axial solid masses with well-defined borders and exhibit strong enhancement following contrast administration. In some cases, they may involve or erode adjacent bone structures [12,13]. While the lesions in our two patients were extra-axial, their radiographic appearance differed from that typically seen in meningiomas, likely due to prior surgical resections.

Comprehensive treatment of intracranial SFTs centers on maximal safe tumor resection. However, for giant lesions, such as those observed in our cases, vascular control is crucial due to the tumor's high vascularity [14]. Therefore, endovascular management through preoperative embolization becomes a fundamental component of hybrid and comprehensive treatment strategies [14,15]. According to Almefty et al., the key to successful treatment lies in the ability to catheterize and embolize small, deep-seated feeding arteries from the internal circulation, which are otherwise difficult or impossible to access surgically in giant tumors [16,17]. Branches of the internal carotid artery can be safely and effectively embolized with the use of advanced endovascular techniques and technology [16]. Both preoperative and intraoperative embolization methods are available. However, endovascular embolization carries inherent risks such as radiation exposure, contrast-induced nephropathy, vascular access complications, infarction due to non-target embolization, and tumor-related edema leading to neurological decline [17,18]. Additionally, there is an increased risk of intracranial hemorrhage, with Carli et al. and Bendszus et al. reporting hemorrhage rates of 5% and 3%, respectively [17,18]. In our cases, preoperative embolization was successfully performed in both patients without any neurological deterioration or intracranial hemorrhage, aligning with the best outcomes described in the literature. Based on our experience, these cases exemplify a multimodal, hybrid, and comprehensive approach to managing giant intracranial solitary fibrous tumors. This validates the concept of hybrid neurosurgery, where endovascular techniques minimize the risk of massive intraoperative blood loss and microsurgical expertise enables maximal safe resection of these highly vascularized tumors.

## Conclusions

Treatment of SFTs with preoperative embolization followed by complete surgical resection is essential due to the highly vascular nature of these tumors and their potential for recurrence and malignant transformation. Preoperative embolization significantly reduces tumor vascularity, thereby minimizing intraoperative blood loss and facilitating safer, more effective resection. Moreover, maximal safe surgical resection remains the cornerstone of treatment, offering optimal local disease control and improving long-term survival. The combination of embolization and complete resection is fundamental to the successful management of SFTs, enhancing surgical outcomes and lowering the risk of recurrence.
